# Health-Related Quality of Life in Systemic Lupus Erythematosus and Its Relationship With Disease Activity: A Single-Center Cross-Sectional Study From Pakistan

**DOI:** 10.7759/cureus.72001

**Published:** 2024-10-21

**Authors:** Tashia Malik, Muneeba Malik, Sumera Ghani, Nauman Ismat Butt, Muhammad Shahid Jamil, Barak Waris

**Affiliations:** 1 Rheumatology/Internal Medicine, Jinnah Postgraduate Medical Centre, Lahore, PAK; 2 Medicine, Sharif Medical City Hospital, Sharif Medical and Dental College, Lahore, PAK; 3 Rheumatology, Kulssom Bai Valika Teaching Hospital, Karachi, PAK; 4 Internal Medicine/Rheumatology, Azra Naheed Medical College, Superior University, Lahore, PAK; 5 Pediatrics, Sharif Medical City Hospital, Sharif Medical and Dental College, Lahore, PAK; 6 Medicine and Allied Studies, Chaudhry Muhammad Akram Teaching and Research Hospital, Azra Naheed Medical College, Superior University, Lahore, PAK

**Keywords:** disease activity, health-related quality of life, sle-specific quality of life (sleqol), systemic lupus erythematosus, systemic lupus erythematosus disease activity index (sledai)

## Abstract

Objective

This study aims to determine whether disease activity was associated with health-related quality of life (HRQoL) in the Pakistani population with systemic lupus erythematosus (SLE).

Methods

This cross-sectional study was conducted in the Rheumatology Department of the National Hospital & Medical Centre, Lahore, Pakistan, from April 2021 to June 2022. A sample of 60 patients, both male and female, diagnosed with a case of SLE was collected through the purposive sampling technique. The data were collected through a questionnaire in which the Systemic Lupus Erythematosus Disease Activity Index (SLEDAI) was applied to assess age at diagnosis, duration, and activity of SLE. HRQoL was evaluated using the Systemic Lupus Erythematosus-Specific Quality of Life (SLEQOL) questionnaire. A seven-digit Likert scale was developed to apply the Student's t-test. The Pearson test was applied to demographic variables.

Results

The mean age of the participants was 32.63 ± 11.12 years. The mean SLEDAI score was 5.30 ± 4.96, ranging from 0 to 17. The mean SLEQOL score was 106.2 ± 33.60, with scores ranging from 47 to 199. There was a significant positive correlation between the SLEDAI score and the overall SLEQOL score (r = 0.376, p = 0.003). Significant correlations were also observed between the SLEDAI score and the subdomains of Physical Functioning (r = 0.341, p = 0.008), Activities (r = 0.478, p < 0.001), Symptoms (r = 0.326, p = 0.011), Treatment (r = 0.262, p = 0.044), and Mood (r = 0.297, p = 0.021). No significant correlation was found between the SLEDAI score and the Self-Image subdomain (r = 0.081, p = 0.541).

Conclusion

High levels of disease, especially fever, pain, and fatigue, cause many obstacles in maintaining life and never help in better understanding the quality of life in terms of physical, psychological, and environmental domains.

## Introduction

Systemic lupus erythematosus (SLE) is a chronic autoimmune disease with a highly varied clinical presentation involving multiple systems of the body and episodes of relapses and remission. Over the past years, life expectancy has increased in SLE patients by understanding the subtleties of the disease together with the improved medical care measures [1]. In the last five decades, with the help of newly standardized scores that have been validated in assessing disease activity and quality of life (QoL), there has been marked improvement in the prognosis of SLE [2]. In addition, a high population of SLE patients had shown poor response and intolerance towards some existing therapies, leading to a negative impact on the QoL [3].

The health-related quality of life (HRQoL) is a multidimensional concept that emphasizes the patient's general perception of the impact of disease or treatment on their health [4]. During the long-term process of the disease, a large number of patients suffer from emotional disturbances with poor sleep quality. The incidence of depression and anxiety among SLE patients ranged from 8.7% to 78.6% and 1.1% to 71.4%, respectively [5]. HRQoL is reduced in patients with SLE as compared to the general population and patients suffering from acquired immune deficiency syndrome (AIDS), Sjögren's syndrome, and rheumatoid arthritis [6]. The prevalence and clinical evolution of SLE have been related to ethnicity, socioeconomic status, and environmental factors, such as poor social status, which is associated with high disease activity and poor mental performance. Misperceptions of the disease and mood disorders due to poor QoL among SLE patients deteriorate the prognosis and treatment. Therefore, evaluation of the health status among SLE patients should incorporate lupus disease activity, damage, and health-related QoL [3,7].

The correlations between HRQoL and disease activity have not been studied in the Pakistani SLE population. The physician's assessments of disease activity and damage do not capture the patient's perspective of their health, and these differences could lead to non-adherence to therapy. Based on that, a comprehensive evaluation of SLE should include the assessment of HRQoL or the sum of the physical, psychological, and social perceptions of well-being influenced by the patient's illness. Therefore, due to the clinical relevance of HRQoL in health disparities and the severity of SLE, this study aimed to determine whether disease activity was associated with HRQoL in the Pakistani population with SLE. The rationale for conducting this study in Pakistan stems from the lack of existing research on the relationship between disease activity and HRQoL in the local SLE population. Understanding this correlation is crucial, as physician assessments often overlook patients' subjective experiences, potentially leading to non-adherence to therapy. Additionally, Pakistan has unique sociocultural factors that may influence disease perception and QoL, making it essential to explore these dynamics in order to improve patient care and tailor interventions to meet the specific needs of this population. By highlighting the patient perspective, this study aims to enhance treatment adherence and overall health outcomes for individuals living with SLE in Pakistan.

## Materials and methods

The present cross-sectional study was conducted on 60 male and female patients diagnosed with SLE at the Rheumatology Department of the National Hospital & Medical Centre, Lahore, Pakistan, from April 2021 to June 2022. Patients were included if they met at least four diagnostic criteria for SLE, were able to communicate verbally or via written consent, and could complete the study proforma. Only those willing to participate voluntarily were enrolled. Patients with suspected or confirmed pregnancy, concurrent infectious diseases, and diagnosed psychiatric illnesses (such as anxiety and depression) were excluded from the study as these conditions may also affect the QoL independent of SLE. A sample size of 60 patients is justified for this study as it serves as a solid foundation for exploratory research, enabling preliminary insights into the relationship between HRQoL and disease activity in SLE. This size allows for meaningful statistical analyses, particularly for medium to large effect sizes, while also balancing practical considerations such as feasibility and resource constraints. Additionally, existing literature supports similar sample sizes, yielding valuable findings and reinforcing the relevance and clinical significance of the results obtained from this study.

A proforma was used for data collection, consisting of questions covering the patient's demographic information and assessing the relationship between SLE and QoL. Six domains of the SLE-specific Quality of Life (SLEQOL) were evaluated: physical function, daily activities, symptoms, mood, treatment, and self-image, through a total of 40 questions [8]. Each item has a seven-point scale ranging from 1 ("not difficult at all," "no trouble at all," or "not often at all") to 7 ("extremely difficult," "extremely problematic at all," or "extremely often") [8]. The sum of the scores ranges from 40 to 280, where high scores indicate poor HRQoL [8]. Disease activity was analyzed using the SLE Disease Activity Index (SLEDAI), categorizing patients based on disease activity, from no activity (<80) to severe activity (>80) [9]. The SLEDAI-2K was obtained from the Mapi Research Trust​ (https://eprovide.mapi-trust.org), which provides licensing and permissions for its use in research. No modifications were made to either tool, and appropriate permissions were obtained from the original distributors to use them in their unmodified forms. Where possible, participants were asked to complete the proforma themselves. However, for those with lower levels of education, the researcher assisted in the form completion while ensuring complete privacy. The researcher's role was strictly to read the questions, allowing participants to indicate their responses. This assistance was provided in an unbiased manner.

The collected data were analyzed using IBM SPSS Statistics for Windows, Version 27 (Released 2020; IBM Corp., Armonk, New York). Categorical variables are presented as frequencies and percentages, while continuous variables are expressed as means and standard deviations (SD). Pearson correlation coefficients were used to compare the SLEDAI and SLEQOL scores. A p-value of less than 0.05 was considered statistically significant in determining the association between QoL domains and clinical variables.

## Results

A total of 60 participants were included in the study, with a predominance of females (n = 55, 91.7%). The age distribution showed that 11.7% (n = 7) were under 20 years old, 66.7% (n = 40) were between 20 and 39 years old, 18.3% (n = 11) were between 40 and 59 years old, and 3.3% (n = 2) were 60 years or older. The mean age of the participants was 32.63 ± 11.12 years, as shown in Table 1. Regarding marital status, 30.0% (n = 18) were single, while 70.0% (n = 42) were married. The majority of patients (n = 45, 75.0%) had higher education, as depicted in Table 1. Employment status indicated that 23.3% (n = 14) were employed, whereas 76.7% (n = 46) were unemployed. Economic status revealed that 20.0% (n = 12) were from the lower class, 50.0% (n = 30) from the middle class, and 30.0% (n = 18) from the upper class. Disease activity, measured by the SLEDAI score, showed that 28.3% (n = 17) had no activity, 26.7% (n = 16) had mild activity, 31.7% (n = 19) had moderate activity, and 13.3% (n = 8) had severe activity, as shown in Table 1.

**Table 1:** Clinical and demographic characteristics of the study participants SLEDAI: Systemic Lupus Erythematosus Disease Activity Index

Characteristics	Frequency (n)	Percent (%)
Gender		
Female	55	91.7%
Male	5	8.3%
Age group (years)		
<20	7	11.7%
20-39	40	66.7%
40-59	11	18.3%
≥60	2	3.3%
Mean age ± SD	32.63 ± 11.12 years	
Marital status		
Unmarried	18	30.0%
Married	42	70.0%
Education		
Primary	5	8.3%
Secondary	10	16.7%
Higher	45	75.0%
Employment status		
Employed	14	23.3%
Unemployed	46	76.7%
Economic status		
Lower class	12	20.0%
Middle class	30	50.0%
Upper class	18	30.0%
SLEDAI groups		
No activity	17	28.3%
Mild activity	16	26.7%
Moderate activity	19	31.7%
Severe activity	8	13.3%

Table 2 summarizes the SLEDAI and SLEQOL scores of the study participants. The mean SLEDAI score was 5.30 ± 4.96, ranging from 0 to 17. The mean SLEQOL score was 106.2 ± 33.60, with scores ranging from 47 to 199. Subscales of the SLEQOL revealed the following mean scores: Physical Functioning, 16.8 ± 6.85 (range 7-35); Activities, 25.5 ± 9.58 (range 9-51); Symptoms, 18.3 ± 6.14 (range 8-39); Treatment, 9.3 ± 4.58 (range 4-25); Mood, 12.3 ± 5.75 (range 4-31); and Self-Image, 24.1 ± 10.15 (range 9-50).

**Table 2:** SLEDAI and SLEQOL scores of the SLE patients SLEDAI: Systemic Lupus Erythematosus Disease Activity Index; SLEQOL: Systemic Lupus Erythematosus-Specific Quality of Life; SLE: systemic lupus erythematosus

Score	Mean ± SD	Range
SLEDAI score	5.30 ± 4.96	0–17
SLEQOL score	106.2 ± 33.60	47–199
QOL: Physical functioning	16.8 ± 6.85	7–35
QOL: Activities	25.5 ± 9.58	9–51
QOL: Symptoms	18.3 ± 6.14	8–39
QOL: Treatment	9.3 ± 4.58	4–25
QOL: Mood	12.3 ± 5.75	4–31
QOL: Self-image	24.1 ± 10.15	9–50

Table 3 shows the Pearson correlation coefficients between the SLEDAI score and the overall SLEQOL score, as well as its subdomains. There was a significant positive correlation between the SLEDAI score and the overall SLEQOL score (r = 0.376, p = 0.003). Significant correlations were also observed between the SLEDAI score and the subdomains of Physical Functioning (r = 0.341, p = 0.008), Activities (r = 0.478, p < 0.001), Symptoms (r = 0.326, p = 0.011), Treatment (r = 0.262, p = 0.044), and Mood (r = 0.297, p = 0.021). No significant correlation was found between the SLEDAI score and the Self-Image subdomain (r = 0.081, p = 0.541).

**Table 3:** Pearson correlation (r) between disease activity and SLEQOL scores *p-value <0.05, significant SLEQOL: Systemic Lupus Erythematosus-Specific Quality of Life

Score	SLEDAI	
	Correlation coefficient (r)	p-value
SLEQOL (overall)	0.376	0.003*
QOL: Physical functioning	0.341	0.008*
QOL: Activities	0.478	<0.001*
QOL: Symptoms	0.326	0.011*
QOL: Treatment	0.262	0.044*
QOL: Mood	0.297	0.021*
QOL: Self-image	0.081	0.541

As presented in Table 4, the SLEQOL scores varied significantly among different SLEDAI groups. Patients with no disease activity had a mean SLEQOL score of 87.6 ± 29.66. Those with mild activity had a mean score of 102.4 ± 33.06, moderate activity had a mean score of 116.7 ± 30.21, and severe activity had a mean score of 128.2 ± 33.01 (p = 0.005). Significant differences were also observed in the subdomains of Physical Functioning (mean ± SD: No activity = 12.9 ± 4.40, Mild activity = 16.5 ± 5.14, Moderate activity = 18.5 ± 7.81, Severe activity = 21.6 ± 8.18; p = 0.020); Activities (mean ± SD: No activity = 19.6 ± 6.90, Mild activity = 24.3 ± 8.91, Moderate activity = 28.2 ± 8.95, Severe activity = 34.1 ± 9.82; p = 0.001); Symptoms (mean ± SD: No activity = 15.1 ± 5.58, Mild activity = 18.8 ± 5.73, Moderate activity = 19.7 ± 6.72, Severe activity = 20.8 ± 4.80; p = 0.034); and Mood (mean ± SD: No activity = 9.8 ± 5.42, Mild activity = 11.3 ± 6.62, Moderate activity = 14.1 ± 5.12, Severe activity = 15.0 ± 3.93; p = 0.016). The Treatment subdomain approached significance (mean ± SD: No activity = 7.8 ± 3.59, Mild activity = 8.7 ± 5.59, Moderate activity = 9.9 ± 3.12, Severe activity = 11.9 ± 6.36; p = 0.008), while no significant difference was found in the Self-Image subdomain (mean ± SD: No activity = 22.4 ± 9.51, Mild activity = 22.8 ± 10.63, Moderate activity = 26.3 ± 9.82, Severe activity = 24.9 ± 12.10; p = 0.549). These findings indicate that higher disease activity is associated with poorer quality of life across several dimensions in SLE patients.

**Table 4:** Mean difference in SLEQOL scores among different groups of disease activity *p-value <0.05, significant **Kruskal-Wallis test SLEQOL: Systemic Lupus Erythematosus-Specific Quality of Life

Score	SLEDAI				p-value**
	No activity (n = 17)	Mild activity (n = 16)	Moderate activity (n = 19)	Severe activity (n = 8)	
	Mean ± SD	Mean ± SD	Mean ± SD	Mean ± SD	
SLEQOL	87.6 ± 29.66	102.4 ± 33.06	116.7 ± 30.21	128.2 ± 33.01	0.005*
QOL: Physical functioning	12.9 ± 4.40	16.5 ± 5.14	18.5 ± 7.81	21.6 ± 8.18	0.020*
QOL: Activities	19.6 ± 6.90	24.3 ± 8.91	28.2 ± 8.95	34.1 ± 9.82	0.001*
QOL: Symptoms	15.1 ± 5.58	18.8 ± 5.73	19.7 ± 6.72	20.8 ± 4.80	0.034*
QOL: Treatment	7.8 ± 3.59	8.7 ± 5.59	9.9 ± 3.12	11.9 ± 6.36	0.008*
QOL: Mood	9.8 ± 5.42	11.3 ± 6.62	14.1 ± 5.12	15.0 ± 3.93	0.016*
QOL: Self-image	22.4 ± 9.51	22.8 ± 10.63	26.3 ± 9.82	24.9 ± 12.10	0.549

## Discussion

The HRQoL in SLE is influenced by disease activity and various psychosocial factors. The impact of disease activity on HRQoL parameters has been extensively studied, revealing a significant association between disease activity and various HRQoL aspects, underscoring the importance of effectively managing disease activity to improve patients' overall well-being [10]. Studies showed that while overall stable disease activity in SLE patients tends to maintain consistent HRQoL levels, even patients achieving low disease activity or remission may still experience poor HRQoL, indicating a gap in current assessment criteria [11,12]. Understanding these relationships can aid in developing tailored interventions to enhance the well-being of SLE patients. Thus, our study aimed to determine whether disease activity was associated with HRQoL in the Pakistani population with SLE.

In our study, the proportion of females (91.7%) was higher compared to male patients (8.3%) with SLE. These results were in agreement with the study conducted by Darvish et al., who reported a higher proportion of females (91%) compared to males (9%) [13]. A study conducted by Gomez et al. also reported a higher proportion of females (94.3%), but it was higher than our study [14]. In the present study, the age of the participants ranged from 13 to 62 years, with the majority falling in the 20-39 years age group (66.7%), and the mean age was 32.6 ± 11.12 years. Chaigne et al. reported that the mean age of the patients in their study was 43 years [15]. Additionally, Hashemi et al. reported that the mean age in their study was 34.09 ± 8.96 years [16].

In the present study, most of the patients were married (70%). These results were in agreement with the study conducted by Hashemi et al., who also reported a higher proportion of married patients (78.57%) [16]. Most patients (76.7%) were unemployed in our study. Similarly, Campbell et al. reported that 92% of the patients in their study had stopped working due to their health problems [17]. Ekblom-Kullberg et al. reported 38% of patients being unemployed, which contrasts with our study [18]. Regarding educational status, 75% of the patients had achieved higher education in our study. George et al. reported that 81% of patients had a high education, which corresponds to our study [19]. In the present study, most of the patients belonged to the middle class (50%), followed by the upper class (20%). Sherby et al. used SLEDAS to determine disease activity and reported a mean SLEDAS score of 105.69 ± 32.37 [20]. Our study showed a positive correlation between disease activity and QoL. Eid et al. also showed a positive correlation between disease activity and QoL [21]. Shi et al. evaluated the factors related to the QoL of SLE patients to show that SLE patients had poor to moderate QoL [22].

SLE is a chronic autoimmune disease characterized by a diverse natural history and multisystem involvement [23]. Over the past five decades, the prognosis for SLE has improved significantly, aided by newly standardized and validated scores for assessing disease activity and QoL. The survival rates have dramatically increased, with the five-year survival rate rising from 5% in 1955 to 95% in 2003 and the 10-year survival rate from 0% to 92%, largely due to earlier diagnosis and optimized treatment strategies [24]. HRQoL is a multidimensional measure that evaluates the impact of health status on overall QoL, typically assessed through various indicators of self-perceived health and physical and emotional functioning. Evaluating HRQoL in patients with chronic conditions can enhance patient management and inform primary care service evaluations. Patients with SLE now have better survival rates compared to previous decades; however, they still report low HRQoL. HRQoL in SLE is significantly impacted by disease activity and psychiatric disorders, particularly anxiety and depression, while socioeconomic status does not appear to influence it. Research using the LupusQoL has shown that various clinical factors, such as disease activity, accumulated damage, fibromyalgia, and mental health issues, play a critical role in determining HRQoL. For instance, a study by Shen et al. found that depression was a major contributor to poorer HRQoL among SLE patients (ß = -0.616, p<0.05), with more depressive symptoms correlating with higher rates of work disability [25]. Etchegaray-Morales et al. recruited 138 women with SLE and demonstrated that poorer HRQoL was significantly associated with depression (r = -0.61; p<0.005), fibromyalgia (r = -0.42; p<0.005), disease activity (r = -0.37; p<0.005), and damage (r = -0.31; p<0.005) [3]. Similarly, Yilmaz-Oner et al. used the SF-36 to show that SLE patients with Hospital Anxiety and Depression Scale (HADS) scores ≥8 had significantly lower HRQoL across all domains (p=0.000) compared to those without anxiety or depression [26]. There are scarce local studies from Pakistan that indicated moderate and severe depression in rheumatoid arthritis patients and cognitive dysfunction in two-thirds of SLE patients. These findings underscore the critical importance of addressing mental health in improving the overall well-being of individuals with SLE.

The strengths of our study on HRQoL in SLE patients from Pakistan are multifaceted. By employing the validated SLEQOL questionnaire, the research provides reliable insights into patient experiences within a culturally relevant context. The focus on HRQoL underscores the importance of patient perspectives in managing chronic diseases, which can inform healthcare strategies and policies tailored to the unique challenges faced by SLE patients in Pakistan. This sets a foundation for future research in this area, ultimately contributing to improved care and resource allocation. The main limitations of our study include a small sample size, single-center design, and reliance on self-reported data. The study's limitations include its small sample size, which may restrict the robustness of findings and their generalizability to the broader SLE population in Pakistan. Additionally, the single-center design may introduce biases that could affect the validity of the results. The reliance on self-reported data could also influence accuracy, as patients' perceptions may be subject to various biases. To address these limitations, future research should prioritize longitudinal and multi-center studies, which can provide a more comprehensive understanding of the relationship between disease activity and HRQoL. Such studies would enable the development of targeted interventions aimed at improving patient outcomes and ensuring that treatment strategies are aligned with patients' needs and experiences.

## Conclusions

Our study highlights the complex interplay between the disease activity of SLE and HRQoL. Indeed, high levels of disease activity, such as fever, pain, and fatigue, can significantly hinder daily functioning and overall well-being. Focusing future research on longitudinal and multi-center studies is crucial. These approaches can provide a more comprehensive view of how disease activity impacts HRQoL over time and across different populations. By exploring these relationships, researchers can identify specific factors contributing to HRQoL declines and develop targeted interventions to improve outcomes. Additionally, integrating patient-reported outcomes with clinical measures could provide deeper insights into how physical, psychological, and environmental factors interact. This could lead to more personalized treatment strategies that address the holistic needs of patients. Overall, emphasis on understanding these dynamics is vital for advancing patient care.

## Appendices

Systemic Lupus Erythematosus specific Quality Of Life (SLEQOL) questionnaire

To obtain the questionnaire, contact the authors (K.P. Leong at Khai_pang_leong@ttsh.com.sg) for the original version [8].

Systemic Lupus Erythematosus Disease Activity Index 2000 (SLEDAI-2K): Data Collection Sheet

Study No.: _____________________ Patient Name: _____________________ Visit Date: _____________________

(Enter weight in the SLEDAI-2K Score column if the descriptor is present at the time of the visit or in the preceding 30 days.)

**Table 5:** Systemic Lupus Erythematosus Disease Activity Index 2000 (SLEDAI-2K) to stratify the severity of systemic lupus erythematosus (SLE)

Weight	Score	Descriptor	Definition
8		Seizure	Recent onset; exclude metabolic, infectious, or drug causes.
8		Psychosis	Altered ability to function in normal activity due to severe disturbance in perception of reality.
8		Organic brain syndrome	Altered mental function with impaired orientation or memory; rapid onset and fluctuating features.
8		Visual disturbance	Retinal changes of SLE; include cytoid bodies, retinal hemorrhages, etc.
8		Cranial nerve disorder	New onset of sensory or motor neuropathy involving cranial nerves.
8		Lupus headache	Severe, persistent headache; nonresponsive to narcotic analgesia.
8		CVA (Cerebrovascular accident)	New onset of cerebrovascular accident(s); exclude arteriosclerosis.
8		Vasculitis	Ulceration, gangrene, tender finger nodules, or proof of vasculitis via biopsy/angiogram.
4		Arthritis	Pain and signs of inflammation in >2 joints (tenderness, swelling, or effusion).
4		Myositis	Proximal muscle weakness/aching with elevated creatine phosphokinase.
4		Urinary casts	Presence of heme-granular or red blood cell casts.
4		Hematuria	>5 red blood cells/high power field; exclude stone, infection, or other causes.
4		Proteinuria	>0.5 grams/24 hours.
4		Pyuria	>5 white blood cells/high power field; exclude infection.
2		Rash	Inflammatory type rash.
2		Alopecia	Abnormal, patchy or diffuse loss of hair.
2		Mucosal ulcers	Oral or nasal ulcerations.
2		Pleurisy	Pleuritic chest pain with pleural rub or effusion.
2		Pericarditis	Pericardial pain with at least 1 confirming sign (rub, effusion, etc.).
2		Low complement	Decrease in CH50, C3, or C4 below lower limit of normal.
2		Increased DNA binding	Increased DNA binding by Farr assay above normal range.
1		Fever	>38°C; exclude infectious cause.
1		Thrombocytopenia	<100,000 platelets/x10^9/L; exclude drug causes.
1		Leukopenia	<3,000 white blood cells/x10^9/L; exclude drug causes.

Total Score: _____________________

*Interpretation:* ​​​​​Activity categories defined on the basis of SLEDAI-2K scores: no activity (SLEDAI=0), mild activity (SLEDAI=1 to 5), moderate activity (SLEDAI=6 to 10), and severe activity (SLEDAI≥11) [9].
